# Restriction of sexual reproduction in the moss *Racomitrium lanuginosum* along an elevational gradient

**DOI:** 10.1002/ece3.6666

**Published:** 2020-08-19

**Authors:** Fumino Maruo, Satoshi Imura

**Affiliations:** ^1^ Department of Biological Sciences Faculty of Science and Engineering Chuo University Bunkyo‐ku Japan; ^2^ National Institute of Polar Research Research Organization of Information and Systems Tachikawa Japan; ^3^ Department of Polar Science School of Multidisciplinary Science SOKENDAI (The Graduate University for Advanced Studies) Tachikawa Japan

**Keywords:** antheridia, archegonia, bryophytes, elevational gradient, gametangia, moss, sexual reproduction

## Abstract

1. Terrestrial plant populations located at the margins of species’ distributions often display reduced sexual reproduction and an increased reliance on asexual reproduction. One hypothesis to explain this phenomenon is that the decline is associated with environmental effects on the energetic costs to produce reproductive organs.
2. In order to clarify the changing processes of sexual reproduction along an elevational gradient, we investigated the sexual reproductive parameters, such as the number of sporophytes and gametangia, in *Racomitrium lanuginosum*, a dioicous moss found on Mt. Fuji.
3. Matured sporophytes were present only below 3,000 m, and the number of sporophytes per shoot tended to be lower at higher elevation habitats. The numbers of male inflorescences per shoot and antheridia per inflorescence and shoot significantly decreased with increasing elevation. In contrast, the numbers of female inflorescences per shoot and archegonia per inflorescence and shoot varied little across elevations.
4. *Synthesis*. Our results suggest that the reasons for this limitation are assumed to be limitations in sporophyte development that result in abortion, and the spatial segregation between males and females. Possible reasons for the abortion of sporophytes are the inhibitory effects of low air temperature, a shortened growth period, and winter environmental conditions at higher elevations. Remarkable differences between male and female on various reproductive parameters found in this study are thought to affect the mode of sexual reproduction under the harsh environment. These differences between males and females may be caused by differences in the costs of production and development of gametangia, sensitivity to environmental stressors, and phenological patterns.

## Graphical abstract

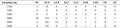

## 1 INTRODUCTION

Reproduction is the basis of the survival and maintenance of species and populations (Ramawat, Mérillon, & Shivanna, 2014). There are two main types of reproductive systems: sexual and asexual reproduction. Each reproductive system includes different productive strategies according to the life history and growth environment of each species (Bengtsson & Ceplitis, 2000; Obeso, 2002).

All plant species have limits to their distribution, and population margins demarcate the limits of adaptation to environmental changes. Terrestrial plant populations located at the margins of species’ distributions often display reduced sexual reproduction and an increased reliance on asexual reproduction (e.g., Eckert, 2002; Pigott, 1981). For example, at the northern limits of the distribution of *Betula glandulosa*, which can reproduce both sexually and asexually, fewer than 0.5% of seeds are viable, and populations are maintained by asexual reproduction (Weis & Hermanutz, 1993). One hypothesis to explain this phenomenon is that the decline is associated with environmental depression on the energetic costs to produce reproductive organs (Fisher, 2011). As cause of restriction of sexual reproduction, environmental/geographical limits of either or both of sexes and geographical/genetic isolation between females and males have been reported (e.g., Pereira, Dambros, & Zartman, 2016; Vitasse et al., 2013). However, the environmental effects were much stronger than genetic effects on sexual reproduction parameters as phenology (Vitasse et al., 2013). Therefore, we focus on environmental/geographical limits of sexual reproduction in the extended area.

Bryophytes are terrestrial atracheophytes and heteromorphic with haploidy dominant plants, that can allow for two different modes of dispersal of genetic material: via haploid gametes and/or diploid zygotes produced by the haploid gametophyte generation (Mable & Otto, 1998). In other words, bryophytes can reproduce both sexually and asexually (e.g., Maciel‐Silva & Pôrto, 2014). The haploid spores (asexual) often have higher dispersal abilities, whereas diploid spores (sexual) tend to have higher survivorship. This may result in a best‐of‐both‐worlds reproductive strategy, where diploid spores expand in established populations and haploid spores colonize new sites. Bryophytes are ideal candidates for mating ecology studies in plants because of their haploid dominated life cycle, diverse sexual strategies, and reliance on asexual reproduction (Pereira et al., 2016). Also, they can disperse by asexual reproduction beyond the limits of sexual reproduction (Fisher, 2011; Longton, 1988). This extended distribution area maintained by asexual reproduction provides interesting opportunities to study the factors restricting sexual reproduction. Several studies on the distribution of sexually reproducing bryophytes have reported a lower frequency of sporophytes toward the distributional limits (Longton, 1988; Longton & Schuster, 1983). Moreover, reproductive success in some species, for example, *Polytrichum alpestre* Hoppe, is known to decline sharply toward the distributional limits (Longton & Greene, 1967). However, there is only limited information on the reproductive parameters of sexual reproduction, such as the frequency of gametangia and sporophytes, around the limits of sexual reproduction and in the extended distribution area.

Mountainous environments are unique, as the physical distances between high and low elevation sites are short, whereas differences in environmental conditions and topography are large (Korpelainen, Jägerbrand, & von Cräutlein, 2012). High elevation environments are characterized by low temperature and long duration of snow cover (e.g., Ortiz, Arista, & Talavera, 2002), as well as increased radiation and wind speed (Körner, 2007). It has been reported that species at high elevations invest more resources in growth than in reproduction (a conservative approach), while species at low elevations tend to invest more resources in reproduction than in growth (von Arx, Edwards, & Dietz, 2006; Hautier, Randin, Stöcklin, & Guisan, 2009). These findings are often related to the conditions at high elevations in the mountains, where low temperature and a long duration of snow cover lead to low productivity (Körner, 2007).

*Racomitrium lanuginosum* (Hedw.) Brid. is a dioicous moss in the family Grimmiaceae that forms vast mats on rock or sand at open sites (e.g., Herzog, 1926; Noguchi, 1988; Tallis, 1958). The species occurs throughout the world, but is commoner at high altitudes and latitudes and in more humid regions. The phenology of this species at same mountain was reported by Maruo and Imura (2016) that the species entire development in about two years. The archegonia developed quickly in early spring, but antheridia took longer to develop from the previous summer. Fertilization occurred in June and July and spore dispersal occurred in June of the following year. The archegonia took 1 months to mature, the antheridia took 7–10 months, and the sporophytes took 10 months. The development of the antheridia and sporophytes stopped during the winter when the site of Mt. Fuji was covered by snow.

As high elevations may represent marginal habitats of sexual reproduction for *R. lanuginosum*, we hypothesized that the ratio of sexual reproduction in population would decrease at high elevations. Also, we hypothesized as the cause to decrease of sexual reproduction that the developmental process of gametangia and/or sporophytes would disturb, the number of sexual organisms such as archegonia, antheridia, and sporophytes would decrease at high elevation habitats according to environmental stresses such as low temperature and snow cover, and limitation on fertilization due to the distance between males and females or the environmental constraints of fertilization process (e.g., Allen & Antos, 1993; Bisang & Hedenäs, 2005; Van der Velde, During, Van de Zande, & Bijlsma, 2001). The aim of this study was to clarify the changing processes of reproductive parameters of sexual reproduction in *R. lanuginosum* along an elevational gradient.

## 2 MATERIALS AND METHODS

### 2.1 Study site

**FIGURE 1:**
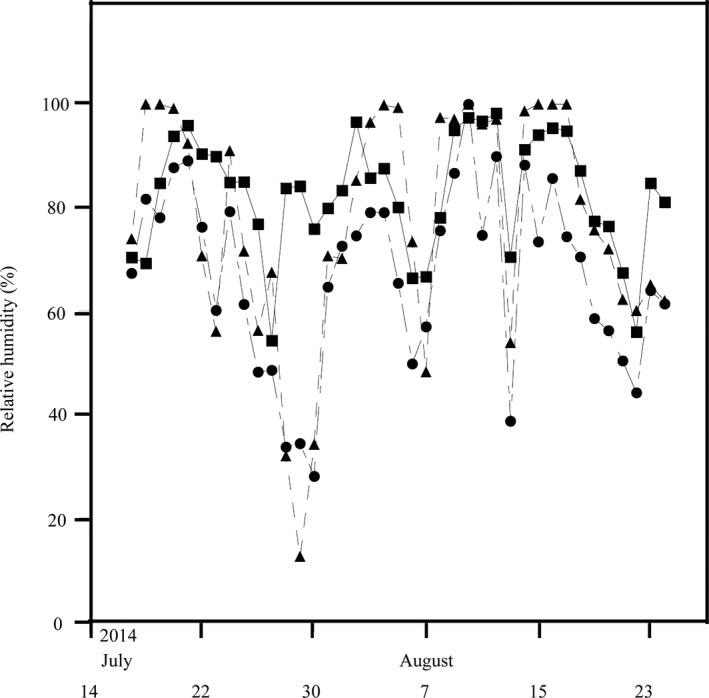
Mean daily relative humidity (%) at ground level at 2,200 (■), 3,100 (●), and 3,700 (▲) m a.s.l. from 17 July to 24 August 2014

Mt. Fuji, the highest mountain in Japan (peak, 3,776 m alt.), is a volcano located in central Honshu (35°21ʹN, 138°43ʹE). In general, the timberline is located at around 2,400–2,500 m alt. (Masuzawa & Suzuki, 1991). The vegetation around the timberline is composed of *Alnus maximowiczii* Callier, *Betula ermanii* Cham., *Salix reinii* French. et Sav. ex Seemen and *Larix kaempferi* (Lamb.) Carrière (Masuzawa, 1985; Sakio & Masuzawa, 1988). The ground in the alpine zone (above the timberline) on this mountain is covered with a thick layer of basaltic scoria formed by past eruptions. In the alpine zone, the slope is patchy and covered with herbaceous perennials, such as *Aconogonon weyrichii* (F.Schmidt) H.Hara var. *alpinum* (Maxim.) H.Hara, *Artemisia pedunculosa* Miq., *Carex doenitzii* Boeck., *Fallopia japonica* (Houtt.) Ronse Decr. var. *japonica*, *Campanula punctate* Lam. var. *hondoensis* (Kitam.) Ohwi, and *Arabis serrta* Franch. et Sav. var. *serrata*. Patches of *A. weyrichii* and *F. japonica* are dominant near the timberline, and *A. serrata* grows at the highest elevation (Masuzawa & Suzuki, 1991). Some studies reported the distributional information of bryophyte in alpine zone on Mt. Fuji such as *Aongstroemia julacea* (Hook.) Mitt., *Barbilophozia sudetica* (Nee ex Huebener) L. Söderstr., De Roo & Hedd., *Bryum argenteum* Hedw., *Ceratodon purpureus* (Hedw.) Brid., and *Diplophyllum albicans* (L.) Dumort. (e.g., Maruo, Furuki, Masuzawa, & Imura, 2019; Takaki, 1971). Populations of *R. lanuginosum* are present on the bare ground at elevations between 1,000 and 3,776 m. (Takaki, 1971). We selected study sites on Mt. Fuji on a northern trail route (the Yoshida trail route) elevation every 200 m between 2,400 and 3,700 m. (Figure 1), except at 2,600 m, at which we could not find an adequate population of *R. lanuginosum*.

### 2.2 Measurements

The mean daily air temperature at ground level at each site was derived from hourly measurements using data loggers (Hobo^@^ Tidbit v2 Part No. UTBI‐001, Onset Computer Corp.) housed in a plastic box at elevation of 2,500, 2,800, 3,000, 3,200, 3,500, and 3,700 m from 17 July 2014 to 28 August 2015. The mean daily relative humidity at ground level was measured by data loggers (Hobo^@^ Pro v2 Part No. U23‐001) at elevation of 2,200, 3,100, and 3,700 m during the summer of 2014.

On 16 July 2014, 10 patches (ca. 100 cm^2^ each) of *R. lanuginosum* were collected randomly by hand or spatula in 25 m^2^ area from each study site and allowed to dry. Each patches were distributed randomly in each site, and the distance between each patches was unstable. Therefore, we paid attention to keep the distance of sampling at least 50 cm. After sampling, every shoot in each patch was dissected. A total of 13,115 shoots were collected from across all sites: 1,529 shoots at 2,400 m, 2,416 shoots at 2,800 m, 1,067 shoots at 3,000 m, 4,167 shoots at 3,200 m, 2,110 shoots at 3,500 m, and 1,826 shoots at 3,700 m. Each shoot was classified by sexual status as male (with antheridia), female (with archegonia or sporophytes), or nonsex expressing (without gametangia and/or sporophytes) under a binocular microscope (Olympus SZ61‐ILST; Olympus). First, the number and developmental stage of sporophytes were observed for all shoots at each study site. Then, 100 shoots were randomly selected from each study site, and the sex ratio of these shoots was recorded. Finally, the number, developmental stage, and size of gametangia were investigated for five randomly selected shoots from each sex from each study site. The developmental stages of the sporophytes and gametangia were identified and described according to Maruo and Imura (2016, 2018).

### 2.3 Statistical analysis

Statistical analyses of the relationships between reproductive parameters and altitude were carried out using R v.3.4.0. (R Core Team, 2017). The *lme* and *glm* function in the *lme4* package were used to implement the linear mixed model and the generalized linear mixed model, respectively.

## 3 RESULTS

### 3.1 Growth environment

The mean annual air temperature decreased with increasing elevation, and the number of days on which the mean daily air temperature was below 0°C at each study site increased with increasing elevation (Table 1). The relative humidity during the summer was similar across the three measured sites (Figure 1).

**TABLE 1:** The mean annual air temperature and number of days on which mean daily air temperature was below 0°C at 2,500, 2,800, 3,000, 3,200, and 3,700 m a. s. l. from July 2014 to June 2015

Elevation (m)	Mean annual air temperature (°C)	Number of days of which mean daily air temperature is below zero (day)
3,700	0.61	227
3,500	0.83	219
3,200	0.42	219
3,000	3.01	218
2,800	2.86	180
2,500	5.88	158

### 3.2 Reproductive parameters

The total percentage of sex expressing shoots was lower than 50.0% at each study site. There were no differences or trends in these percentages along the elevational gradient (Table 2).

**TABLE 2:** Number of male, female, and nonsex expressing (asexual) shoots and sex ratio (%) at each study site on Mt. Fuji

Elevation (m)	Number of shoots (sex ratio (%))			
	Male	Female	Asexual	Total
3,700	12 (12.0)	30 (30.0)	58 (58.0)	100 (100.0)
3,500	– (0.0)	44 (48.9)	46 (51.1)	90 (100.0)
3,200	15 (50.0)	– (0.0)	15 (50.0)	30 (100.0)
3,000	13 (14.9)	26 (29.9)	48 (55.2)	87 (100.0)
2,800	25(25.0)	8 (8.0)	67 (67.0)	100 (100.0)
2,400	20 (20.0)	14 (14.0)	66 (66.0)	100 (100.0)

Sporophytes were found at elevation of 2,400, 2,800, 3,000, and 3,700 m (Table 3). At 3,700 m, only the swollen venter (SV) and early calyptra in perichaetium (ECP) stages were present. Matured sporophytes [empty and fresh (EF) stages] were found below 3,000 m, with most found at 2,400 m (Table 3). As for the number of sporophytes per shoot, this tended to decrease with increasing elevation, and however, there were no significate differences along an elevational gradient (Table 4). At every site, the most abundant developmental stage was ECP (Table 3).

**TABLE 3:** Developmental stages of sporophytes at each study site on Mt. Fuji on 16 July 2014. SV–EF: Developmental stages of sporophytes described in Maruo and Imura (2016, 2018)

Elevation (m)	SV	ECP	LCP	ECI	LCI	EOI	LOI	OF	EF
3,700	5	38	0	0	0	0	0	0	0
3,500	0	0	0	0	0	0	0	0	0
3,200	0	0	0	0	0	0	0	0	0
3,000	29	316	6	17	2	1	3	0	1
2,800	17	102	53	4	0	1	0	0	1
2,400	9	193	0	2	0	1	4	0	33

**TABLE 4:** Numbers of shoots, sporophytes, sporophytes per shoot, and male inflorescences, antheridia, female inflorescences, and archegonia at each study site on Mt. Fuji (*n* = 5)

Elevation (m)	Number of all shoots	Number of all sporophytes	Number of sporophyte per shoot	Total number of male inflorescences	Total number of antheridia	Total number of female inflorescences	Total number of archegonia
3,700	1,826	43	0.024	26	103	10	30
3,500	2,110	0	0	0	0	51	190
3,200	4,167	0	0	35	120	0	0
3,000	1,067	375	0.352	30	139	8	25
2,800	2,416	178	0.074	58	458	35	81
2,400	1,529	242	0.158	56	436	10	56

The numbers of male inflorescences per shoot (glm, *p* = .018), antheridia per shoot (glm, *p* < 2 × 10^–16^), and antheridia per inflorescence (glm, *p* = .003) significantly decreased with increasing elevation (Tables 4 and 5). In contrast, there were no significant differences in the numbers of female inflorescences per shoot (glm, *p* = .573), archegonia per shoot (glm, *p* = .817), or archegonia per inflorescence (glm, *p* = .204) according to elevation (Tables 4 and 5).

**TABLE 5:** Parameter estimates for lme and glm [with Poisson (p) and Gaussian (g) distributions] fitted to the parameters for males and females along an elevational gradient on Mt. Fuji

Parameter	Estimate	Std. error	*Z* or *t* value	Pr (>|*t*|)
Male				
Number of inflorescences per shoot (p)	−0.004 × 10^–1^	0.002 × 10^–1^	−2.377	0.018*
Number of antheridia per shoot (p)	−1.098 × 10^–3^	7.531 × 10^–5^	−14.570	<2 × 10^–16^***
Number of antheridia per inflorescence (g)	−0.004	0.001	−3.260	0.003**
Size of antheridia	−2.833 × 10^–5^	2.946 × 10^–5^	−0.961	0.347
Female				
Number of inflorescences per shoot (g)	0.001	0.002	0.571	0.571
Number of archegonia per shoot (g)	0.002	0.009	0.234	0.234
Number of archegonia per inflorescence (g)	−0.002 × 10^–1^	0.002 × 10^–1^	−1.302	0.200
Size of archegonia	−1.013 × 10^–5^	2.825 × 10^–5^	−0.358	0.724

Signif. codes: 0 ‘***’ .001 ‘**’ .01 ‘*’ .05 ‘.’ .1 ‘ ’ 1.

In terms of shoot size, no significant differences were observed between males and females (glm, *p* = .922) or along an elevational gradient (glm, *p* = .449). The sizes of antheridia (lme, *p* = .347) and archegonia (lme, *p* = .724) were almost identical at each study site, and there was no trend along the elevational gradient (Table 5). The maturation ratio of antheridia was 100.0% at 2,400, 3,200, and 3,700 m; 89.7% at 3,000 m; and 93.1% at 2,800 m. (Table 6). The maturation ratio of archegonia was 100.0% at 2,400, 3,000, 3,500, and 3,700 m and 96.2% at 2,800 m. (Table 6).

**TABLE 6:** Development and maturation ratios of antheridia (An) and archegonia (Ar) at each study site on Mt. Fuji. J–D: Developmental stages of gametangia described in Maruo and Imura (2016, 2018) (*n* = 5)

Elevation (m)		Number of antheridia or archegonia				Maturation ratio (%)
		J	I	M	D	
3,700	An	0	0	2	22	100.0
	Ar	0	0	5	5	100.0
3,500	An	–	–	–	–	–
	Ar	0	0	0	50	100.0
3,200	An	0	0	0	25	100.0
	Ar	–	–	–	–	–
3,000	An	3	0	0	26	89.7
	Ar	0	0	0	6	100.0
2,800	An	1	3	3	51	93.1
	Ar	0	1	5	20	96.2
2,400	An	0	0	0	56	100.0
	Ar	0	0	0	10	100.0

## 4 DISCUSSION

Maruo and Imura (2016) reported a high frequency of matured sporophytes (OF and EF stages) produced at 2,200 m a. s. l. on Mt. Fuji. In this study as alpine zone (2,400 m–3700 m a.s.l.), aborted sporophytes were mainly found. The frequency of matured sporophytes was very low, and matured sporophytes were present only below 3,000 m and mainly found at 2,400 m, which is the lowest elevation in the alpine zone. While the number of sporophytes per shoot at higher elevations tended to be lower than that at lower elevations (Table 4), there were no significate differences along an elevational gradient. This suggested that the success of sexual reproduction in as spore dispersal is restricted only at lower elevations along an elevational gradient and that the environmental conditions in the alpine zone of Mt. Fuji are not suitable for the sexual reproduction of *R. lanuginosum*. Ortiz et al. (2002) reported that in *Juniperus communis* subsp. *alpina* (Suter) Ĉelak., cone reproduction and productive success decreased toward elevational distribution limits. Holm (1994) reported that the number of seeds per catkin decreased with increasing elevation in *Betula pendula* Roth and *B. pubescens* Ehrh. ssp. *pubescens*. Hegazy, Hammouda, Lovett‐Doust, and Gomaa (2008) found that the average number of seeds per individual in *Moringa peregrina* (Forssk.) Fiori significantly decreased along an elevational gradient. The reasons of the restriction of sexual reproduction as sporophyte production are assumed to be: (1) limitation on sex expression; (2) limitation on production and development of gametangia; (3) limitation on fertilization due to the distance between male and female individuals or the environmental constraints of fertilization process, such as a lack of liquid water; (4) limitation on production and development of sporophyte; (5) phenology; and (6) limitation of reproductive resources and genetic differentiation.

### 4.1 Limitation on sex expression

Sex expression is the key factor limiting sporophyte production, as the presence of both male and female individuals is essential for the success of fertilization. Populations of dioicous species sometimes show biased sex ratios, spatial separation of sexes, or the failure of sex expression in males (e.g., Bowker, Stark, McLetchie, & Mishler, 2000; Gemmell, 1950). In extreme environments, the difference of cost allocation in developmental stage of reproductive organs between females and males leads to higher mortality rate in female and resulting male‐biased sex ratios (e.g., Allen & Antos, 1993). In bryophyte populations, sex ratios are predominately female‐biased (Stark, McLetchie, & Eppley, 2010). In this study, the sex ratio at each elevation varied widely. However, a male‐biased sex ratio was not observed, and there was no relationship between sex ratio and elevation detected.

### 4.2 Limitation on production and development of gametangia

Most of the gametangia of this species reached maturity at every elevation, suggesting that environmental factors in the alpine zone did not restrict the development of gametangia. On the other hand, we found interesting differences in the number of gametangia between males and females along an elevational gradient. The number of male inflorescences per shoot, and antheridia per shoot and inflorescence significantly decreased with increasing elevation. In contrast, the number of female inflorescences per shoot and archegonia per shoot and inflorescence were virtually identical across the elevational gradient. This suggests that the production of gametangia results in different responses between males and females along an elevational gradient. This difference may be due to the different costs of the production and development of gametangia between males and females. Compared with female gametangia, the production of male gametangia is more energetically expensive for bryophytes (Stark, McLetchie, & Mishler, 2005; Stark, Mishler, & McLetchie, 2000). In addition, males are reported to be more sensitive to environmental stressors (Bisang & Hedenäs, 2005; Cameron & Wyatt, 1990; Longton, 1985, 1988; Shaw, Jules, & Beer, 1991). The results of this study are therefore consistent with these reports on bryophytes. Consequently, our results suggested that males and females of this species have different strategies with respect to balancing sexual reproduction and survival on marginal high elevation habitats: Males appear to invest energy in gametangial production, while females produce gametangia, but invest less energy than males to support the development of sporophyte (Stark et al., 2005). The number of gametangia has also been proposed to be an important factor in guaranteeing sufficient sperm for fertilization (e.g., Glime, 2007). It has been suggested that the decrease in the number of male gametangia is likely to limit fertilization at higher elevations than this study sites.

### 4.3 Limitation on fertilization

Only male shoots were found at 3,200 m, and only female shoots were observed at 3,500 m, and sporophytes were not produced at these elevations. Limitations on fertilization are thought to arise from the spatial segregation of male and female sex expression, since fertilization depends on sperm mobility (Van der Velde et al., 2001). Some previous studies have reported that the distance that sperm can disperse is on the order of centimeters to meters (Longton, 1976; Van der Velde et al., 2001). In this study, spatial segregation of sex expression between males and females was observed at 3,200 and 3,500 m. This indicates that the males and females are separated by more than several meters at these sites. Consequently, it is thought that the spatial segregation of sex expression between males and females at these sites has a detrimental effect on fertilization and the failure of production.

### 4.4 Limitation on production and development of sporophyte

At every elevation in the alpine zone, the frequency of aborted sporophytes (younger than the OF stage) was very high. Most aborted sporophytes were found to be in the ECP stage at every elevation. This suggested that the ECP stage is an obstacle to maturity. Based on the above results, environments at higher elevations, such as low air temperature and shortened growth periods, are suggested to have a detrimental effect on the production and development of sporophytes.

### 4.5 Phenology

**FIGURE 2:**
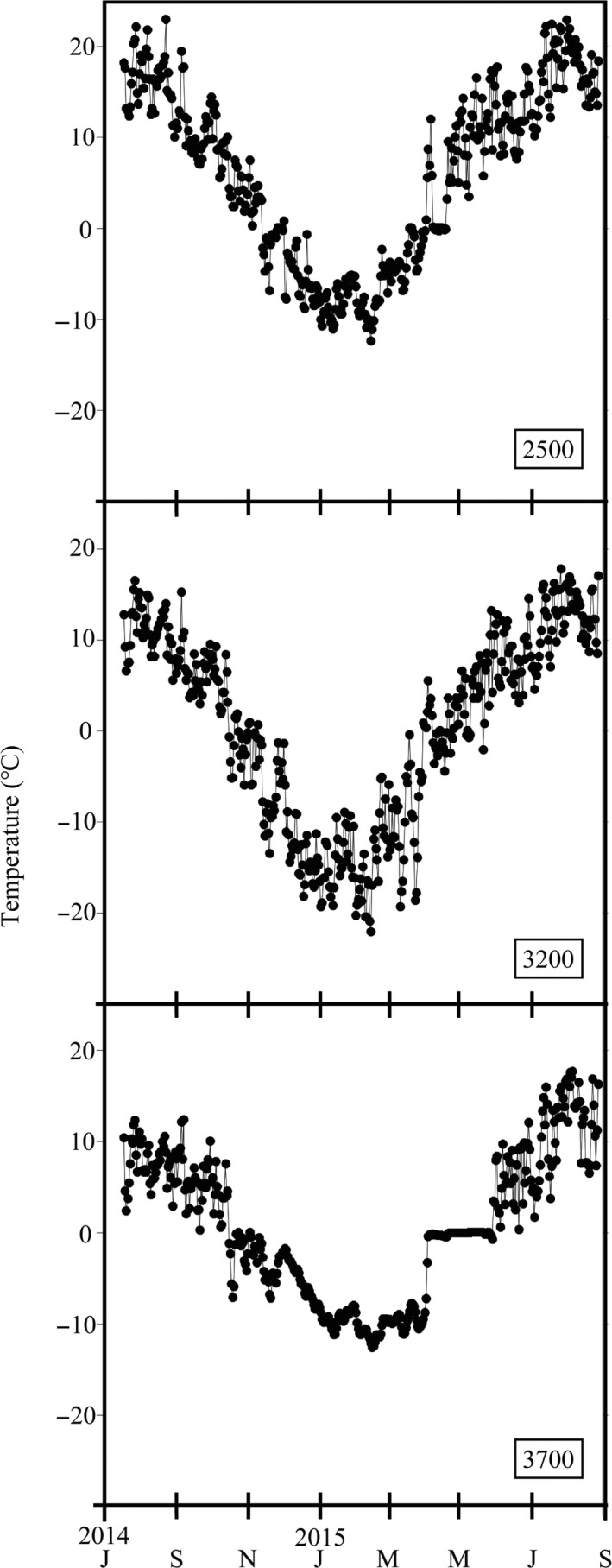
Mean daily air temperature (°C) at ground level at 2,500, 3,200, and 3,700 m a.s.l. from 17 July 2014 to 28 August 2015

Maruo and Imura (2016) reported different phenological patterns in males and females at 2,200 m a.s.l., finding that the duration of male gametangia development was longer, continuing from summer to spring of the following year and including a resting period of development under the snow cover in winter. In contrast, female gametangia developed rapidly in spring and showed a shorter developmental duration than males. Sporophytes emerged in the rainy season and developed from summer to spring of the following year, including a resting period of development under the snow cover, with maturation in the spring. At 2,200 m, sporophytes emerged in the rainy season and developed from summer to spring of the following year, including a resting period of development under the snow cover in winter, with maturation in the spring (Maruo & Imura, 2016, 2018). They have also reported that the air temperature under the snow cover is ca. 0°C, and the relative humidity is ca. 100%. This suggests that snow cover protects the population of *R. lanuginosum* from extreme winter temperatures and desiccation. Some studies have indicated that snow cover shortens the growth period but plays an important role in protecting the plant canopy from winter temperature and desiccation (e.g., Billings & Bliss, 1959; Holway & Ward, 1963). In this study, the winter air temperature at each elevation fluctuated from –15 to –5°C (Figure 2). This indicates that the snow cover at each elevation in the alpine zone was thinned by strong winds and the sloping ground and that populations of *R. lanuginosum* were not protected from harsh winter conditions. This suggested that the development of male gametangia and sporophytes may be affected by low air temperatures throughout the year and desiccation in winter. According to the phenological patterns at 2,200 m (Maruo & Imura, 2016, 2018), sporophyte matured until EOI stage before winter, but we found mainly ECP stage sporophyte on alpine zone, and only SV and ECP stages at 3,700 m. This suggested that the phenological pattern of sporophyte on alpine zone was delayed than 2,200 m due to low productivity caused by low air temperature. The shortened growth period in the alpine zone is assumed to be another reason for the restriction of male gametangia and sporophyte development. The length of the growth period is thought to be restricted by several environmental factors, such as air temperature and water availability. In this study, the number of days on which the mean daily air temperature was below 0°C increased along an elevational gradient. The length of the growth period, that is, the number of days on which the mean daily air temperature was above 0°C, was ca. 6.9 months at 2,500 m and 4.6 months at 3,700 m. Therefore, the growth period at 3,700 m was ca. 1.3 months shorter than that at 2,500 m. Thus, the length of the growth period was reduced and the environment became less suitable for plant growth at increasing elevations. According to the phenological patterns of *R. lanuginosum* at 2,200 m (Maruo & Imura, 2016, 2018), male gametangia require a longer developmental duration to reach maturity than female gametangia. This indicates that a shortened growth period may have a greater negative impact on male gametangia development than on female gametangia development. Consequently, we speculate that the occurrence and development of male gametangia at higher elevation habitats are restricted due to low air temperature, winter conditions, and a shortened growth period. The phenological correspondence of maturation timing between male and female gametangia has also been considered a key factor in the success of fertilization (Maruo & Imura, 2018). According to the number of days on which the mean daily air temperature is below 0°C, it is assumed that there is a shorter growth period at higher elevations. Thus, in this study, the negative impact of the shortened growth period on the phenology of sporophytes and male gametangia has already considered. In addition, a shift in the maturation timing of male gametangia induced by a phenological change due to the shortened growth period may occur. This change in the maturation timing of male gametangia is assumed to cause a discordance in the maturation timing between males and females, resulting in a low frequency of fertilization. Consequently, the phenological patterns of *R. lanuginosum* male gametangia and sporophytes may be another reason for limitations on sporophytes and male gametangia and along an elevational gradient.

### 4.6 Limitation of reproductive resources and genetic differentiation

Also, we assumed that another possible cause is the decrease of photosynthetic productivity due to low temperatures and shortened growth period. A fundamental assumption of life history is that sexual reproduction incurs costs, due to trade‐off between present reproductive investment and future growth, survival, and/or reproductive capacity of the reproducing individuals (e.g., Reznick, Nunney, & Tessier, 2000; Rydgren & ØkLand, 2003). The physiological cause of reproductive costs is assumed to be high expenditure of resources and energy by the reproducing individuals which, in turn, negatively affects their future growth and development (Ehrlén, 1997). Kallio and Heinonen (1973) reported that the optimum temperature for photosynthesis in *R. lanuginosum* is 5°C, within the limits of –10 to 30°C. In this study, air temperature decreased and the length of the growth period was reduced with increasing elevation. Mean annual air temperatures were below 5°C at elevations above 2,800 m. These results suggest that sporophyte development was restricted and the phenology of this species was delayed due to the low productivity caused by the reductions in air temperature and the growth period.

We assumed that the cause of the restriction of sexual reproduction such as the failure of development on sporophytes and the decrease of male gametangia is genetic factor that genotype such as biased genotype toward clone expression, and low productivity of sexual reproductive organs. Genetic diversity within and among populations and phenotypic plasticity of phenological key events, such as sex expression, the emergence of sexual organs, play crucial roles in adaptation (e.g., Vitasse et al., 2013). Bryophytes can keep the high genetic diversity by capability of dispersing long distances of reproductive propagules by wind. In this study, we found the sex expression of one sex at 3,200 and 3,500 m. This suggests that the possibility of the immigration on alpine zone from low elevations and/or other habitats is low, and clonality increases with increasing elevation/environmental stress. On the other hand, we found sex expression of both sex at the highest study site at 3,700 m. We assumed this inconsistent phenomenon at 3,700 m was caused by buffering effect of snow cover during the winter, because the temperate at 3,700 m during winter was more stable and higher than that of 3500/3200 m (Figure 2). Snow cover was kept constantly at 3,700 m in the winter, because the site is flat terrain located on summit of Mt. Fuji, whereas the slope terrain at 3,500/3,200 m would prevent snow accumulation.

In conclusion, the success of sexual reproduction of *R. lanuginosum*, as measured by the production of matured sporophytes and dispersal of spores, was restricted at higher elevation habitats in the alpine zone on Mt. Fuji. Reasons for this limitation are assumed to be limitations in sporophyte development that result in abortion, and the spatial segregation between males and females. Possible reasons for the abortion of sporophytes are the inhibitory effects of low air temperature, a shortened growth period, and winter environmental conditions at higher elevations. Remarkable differences between male and female on various reproductive parameters found in this study are thought to affect the mode of sexual reproduction under the harsh environment. We speculate that these differences are caused by differences in the costs of production and development of gametangia, sensitivity to environmental stressors, and phenological patterns.

## CONFLICT OF INTEREST

None declared.

## AUTHOR CONTRIBUTION

**Fumino Maruo:** Conceptualization (lead); Data curation (lead); Formal analysis (lead); Funding acquisition (lead); Investigation (lead); Methodology (lead); Project administration (lead); Validation (lead); Visualization (lead); Writing‐original draft (lead); Writing‐review & editing (lead). **Satoshi Imura:** Conceptualization (supporting); Data curation (supporting); Formal analysis (supporting); Funding acquisition (supporting); Investigation (supporting); Methodology (supporting); Project administration (supporting); Supervision (lead); Validation (supporting); Visualization (supporting); Writing‐original draft (supporting); Writing‐review & editing (supporting).

## Data Availability

All data generated or analyzed during this study are included in this published article.
